# Clinical trial of laronidase in Hurler syndrome after hematopoietic cell transplantation

**DOI:** 10.1038/s41390-019-0541-2

**Published:** 2019-08-21

**Authors:** Lynda E. Polgreen, Troy C. Lund, Elizabeth Braunlin, Jakub Tolar, Bradley S. Miller, Ellen Fung, Chester B. Whitley, Julie B. Eisengart, Elise Northrop, Kyle Rudser, Weston P. Miller, Paul J. Orchard

**Affiliations:** 1 University of Minnesota Masonic Children’s Hospital, Minneapolis, MN USA; 2 Los Angeles Biomedical Research Institute at Harbor-UCLA Medical Center, Torrance, CA USA; 3 Children’s Hospital Oakland Research Institute, Oakland, CA USA; 4 Division of Biostatistics, University of Minnesota, Minneapolis, MN USA; 5 Sangamo Therapeutics, Richmond, CA USA

## Abstract

**Background:**

Mucopolysaccharidosis I (MPS IH) is a lysosomal storage disease treated with hematopoietic cell transplantation (HCT) because it stabilizes cognitive deterioration, but is insufficient to alleviate all somatic manifestations. Intravenous laronidase improves somatic burden in attenuated MPS I. It is unknown whether laronidase can improve somatic disease following HCT in MPS IH. The objective of this study was to evaluate the effects of laronidase on somatic outcomes of patients with MPS IH previously treated with HCT.

**Methods:**

This 2-year open-label pilot study of laronidase included ten patients (age 5–13 years) who were at least 2 years post-HCT and donor engrafted. Outcomes were assessed semi-annually and compared to historic controls.

**Results:**

The two youngest participants had a statistically significant improvement in growth compared to controls. Development of persistent high-titer anti-drug antibodies (ADA) was associated with poorer 6-min walk test (6MWT) performance; when patients with high ADA titers were excluded, there was a significant improvement in the 6MWT in the remaining seven patients.

**Conclusions:**

Laronidase seemed to improve growth in participants <8 years old, and 6MWT performance in participants without ADA. Given the small number of patients treated in this pilot study, additional study is needed before definitive conclusions can be made.

## Introduction

The mucopolysaccharidoses (MPS) are a group of diseases resulting from specific lysosomal enzyme deficiencies that result in accumulation of glycosaminoglycans. Debilitating disease-related pathology is a significant problem that persists even following standard treatment for Hurler syndrome, the severe form of mucopolysaccharidosis type I (MPS IH). Hurler syndrome is the most severe form of alpha-l-iduronidase (IDUA) deficiency and is characterized by progressive multisystem involvement including severe cognitive, cardiac, pulmonary, and orthopedic complications. Most untreated children with MPS IH die from cardiopulmonary disease by late childhood. The standard of care for MPS IH, hematopoietic cell transplantation (HCT), has been successful in decreasing mortality from cardiac, pulmonary disease, and preserving central nervous system (CNS) function.^1,2^ Consequently, children with MPS IH treated with HCT are living into adulthood.^2,3^ HCT does not, however, adequately treat all disease manifestations in MPS IH. Even in the setting of complete hematopoietic engraftment from enzyme-normal donors, patients with MPS IH treated with HCT are continuing to develop severe orthopedic disease, which may include carpal tunnel syndrome, progressive kyphosis, scoliosis, hip dysplasia, genu valgum, cervical instability, and cord compression, along with cardiomyopathy and valve disease, corneal clouding, sleep disordered breathing, hearing loss, and endocrinological disease.^3–9^ Thus, there is an urgent need for augmentative treatments in patients with MPS IH, despite “successful” transplantation.

Engraftment of allogeneic hematopoietic cells provides a low level of continuous enzyme delivery within tissue and plasma compartments. In contrast, weekly IV laronidase, with a short half-life ranging from 1.5 to 3.6 h (Package Insert), provides intermittent supraphysiologic plasma levels of enzyme. Therefore, each treatment approach has different pharmacokinetics. Enzyme replacement therapy (ERT; (laronidase)) is approved for patients with MPS I, as this therapy is effective in the treatment of most of the somatic manifestations of the disease; however, it does not treat the CNS pathology and is associated with poorer long-term survival for patients with Hurler syndrome.^10^ ERT is routinely used in infants awaiting HCT in order to improve clinical status prior to HCT and prevent pulmonary complications during HCT,^11^ but HCT remains the standard treatment for MPS IH because it can stabilize cognition and prevent progressive, unrecoverable developmental deterioration.^3,12–22^ Administration of laronidase for 8–14 weeks before and shortly following HCT appears to improve IQ, along with nonverbal problem solving and processing at 2 years post-HCT.^16^ In addition, a retrospective, multicenter, long-term follow-up study of 217 patients with MPS IH after treatment with HCT found that a below normal post-HCT IDUA leukocyte enzyme level is associated with increased morbidity, including more progression of cord compression, cervical instability, hip dysplasia, carpal tunnel, overnight hypoxia, and corneal clouding.^3^ Finally, in a murine model of MPS IH, the delivery of supraphysiologic IDUA following transplantation with lentiviral-mediated genetically corrected hematopoietic stem cells resulted in improved appearance of the bone and epiphysis, suggesting a dose effect of enzyme in modifying orthopedic-related outcomes.^23^ Therefore, we hypothesized that the addition of intermittent supraphysiologic dosing of laronidase may benefit patients with MPS IH after HCT. If laronidase, continued after HCT, prevented complications of this disease, this has the potential to significantly improve the lives of affected patients and their families, and may decrease the risk of medical and surgical interventions required to manage disease-related complications. The primary objective of this pilot study was to evaluate safety and tolerability of weekly IV laronidase in patients with MPS IH treated with HCT at least 2 years prior. Secondary objectives evaluated the effect of weekly IV laronidase on joint range-of-motion (ROM), muscle strength, fitness, cardiac findings, and physical activity level.

## Methods

### Patients and screening

Inclusion criteria included patients diagnosed with MPS IH that were <14 years old at study enrollment. Eligible patients must have been treated with HCT > 2 years prior to study enrollment and had >10% donor engraftment. Exclusion criteria included previous administration of laronidase within 2 years, anticipated survival less than 2 years, or history of cardiac or pulmonary insufficiency defined by an ejection fraction <40% or requiring continuous supplemental oxygen, respectively. Subjects were enrolled between June 2012 and February 2014 at the University of Minnesota.

For height and ROM outcomes, the study cohort was compared against historic MPS IH control data from a 9-year longitudinal observational study.^24–26^ Inclusion criteria for the longitudinal study were biochemical and/or genetic diagnosis of MPS IH, age ≥5 years old and <18 years old. Exclusion criteria included concurrent enrollment in a therapeutic drug trial. All of the patients from this control cohort had previously undergone HCT. The historical controls were selected by having MPS IH, history of HCT, and with comparable age at entry into the study.

Informed consent was obtained from all parents/guardians and assent was obtained from subjects when appropriate. The University of Minnesota Human Subjects Committee approved the protocol for this pilot study. The prior observational study was conducted at three sites: Los Angeles Biomedical Research Institute, the University of Minnesota, and Children’s Hospital Oakland Research Center, and was approved by the Human Subjects Committee at each of these institutions. This study was registered on clinicaltrials.gov (NCT 01173016).

### Design

This was an open-label, single arm, 2-year, pilot study of weekly laronidase in subjects with MPS IH who were treated with HCT > 2 years prior to study enrollment. Subjects received weekly laronidase (0.58 mg/kg i.v.) and were monitored for infusion-related toxicities by their local treating physician. The local physicians completed a laronidase administration report form at the time of each infusion and returned the logs to the study coordinators at least every 3 months. Donor hematopoietic chimerism was measured at baseline and every 6 months for the duration of the 2-year study. Antidrug antibodies (ADA) were measured by two methods^27,28^; inhibitory capacity was measured at ADA titer peak and end-of-study as previously described.^28^ Subjects were labeled ADA+ if their maximum titer was ≥4000 (using either method) and the mean inhibition (averaged between the inhibition at the ADA titer peak and the end of the study) was ≥30% as previously described.^28^ Measurements of ROM, muscle strength, fitness, physical activity, and growth were also conducted at baseline and every 6 months. In addition, annualized growth velocity at baseline was calculated using the most recent height measurement obtained in clinical practice, but at least 6 months prior to the baseline visit, in seven of ten participants (data unavailable in three participants).

### Outcome measures

The primary outcome measure was safety and tolerability. Secondary outcome measures included changes from baseline in several key domains. Bilateral shoulder flexion, elbow extension, and hip extension were measured using goniometry and the sites were chosen based on common areas of MPS-related disease. Muscle strength was measured by a Biodex System 3 dynamometer (Biodex medical Systems, Inc., Shirley, NY). The angular velocities of 90 and 120 degrees/second (d/s) were chosen based on previously reported deficits in these strength measures in MPS IH.^24^ Handgrip strength was measured three times in both hands with a mechanical hand-held dynamometer with the subject in a seated position at each visit; the average for each hand is presented. Fitness was measured by peak oxygen uptake (VO_2_ peak), peak heart rate (HR), and time to fatigue during a modified Balke Treadmill Test.^29^ This test is recommended for unfit and/or chronically ill children or adolescents. Briefly, patients began walking at 2.0 mph with a 2% increase in grade every 2 min. A 12-lead electrocardiogram was monitored continuously throughout the test for the determination of heart rate and dysrhythmias or ischemic changes. Heart rate was measured at the end of each stage (i.e. every 2 min) and time to fatigue (i.e. patient request to stop despite encouragement to continue) was recorded. VO_2_ peak was measured by collection and analysis of expired air by a MedGraphics CPX/D Metabolic System. The 6MWT was conducted as previously described.^30^ In brief, a 30 m hospital corridor marked by colored tape at each end was used. Subjects were instructed to walk from end to end at their self-selected pace, while attempting to cover as much distance as possible in the 6 min. The patients were instructed to walk around the mark as they changed direction. The time and distance covered was recorded, as was the heart rate prior to and immediately after completion of the walk test. The 6MWT has previously been used in assessments of laronidase and other ERT interventions in patients with lysosomal storage diseases as a measure of overall physical function and health status.^31–33^ The 6MWT is a measure of overall physical function and health status because performance of this test incorporates multiple body systems. The ability to understand and follow the instructions for the test requires a degree of cognitive ability. The physical performance of the test is a measure of mobility and coordination as well as cardiorespiratory status. As such, this test assesses many functions required to perform typical activities of daily living. Anthropometric measurements (standing height, sitting height, and arm span) were performed in triplicate and the average taken at the baseline and month 24 visits. Height standard deviation scores (SDS) were calculated based on CDC growth charts.^34^ Cardiac ultrasounds were obtained at baseline and month 24. Two-dimensional imaging was obtained for determination of anatomy. Left ventricular chamber dimensions and wall thicknesses were measured by M-mode and shortening fraction (SF [normal > 27%]) was calculated by standard methodology.^35^ Pulse-wave and color Doppler interrogation of cardiac valves was performed for determination of valve regurgitation or stenosis. To compare chamber dimensions and wall thicknesses sequentially in a single subject or between subjects of different sizes, *Z*-scores based upon body surface area were calculated.^35^ A *Z*-score of 0 represents the normal, or average, value at a given body surface area and *Z*-scores between −2 and +2 are values within 2 standard deviations (SD) of normal (parameterz.com).

### Statistical analysis

Data are presented in both a descriptive and quantitative fashion due to the small sample size of this pilot study. The sample size was chosen based on feasibility given the small population size from which to recruit. Linear mixed models with random intercept were used to test for difference in the change in outcome over time between the treated group and the historic control group. An additional identical analysis was performed restricted to treated and control participants under 8 years of age. Linear regression was used to test for associations of age at study entry with change in each outcome, and logistic regression for differences in response by anti-ERT antibody status. Female participants with a bone age >14 years and males with a bone age >16 years during the study were excluded from the growth analysis due to near closure of growth plates. All analyses and graphing were performed with the use of Prism 7 (GraphPad Software, Inc.), R v3.4.0,^36^ and STATA 14. Outcomes are reported as change from the earliest study visit to the last study visit with a minimum of 12 months between measurements. All analyses were pre-specified, except the test for associations between outcomes and age at study entry, which was formally assessed after the initial review of the study results.

## Results

### Study participants

Eleven participants (60% male) were enrolled and followed in the study from June 2012 to April 2016. One withdrew at month 6 at parents’ request and the remaining ten completed the study. Baseline characteristics for these ten are described in Table 1. The rate of adherence to the schedule of weekly infusions of laronidase in each patient ranged from 79 to 100% (median = 99%). There were no reported drug-related severe adverse events.

**Table 1:** Baseline characteristics of participants

Pt#	Prior ERT?	Sex	Age HCT (m)	HCT graft	Age study entry (y)	ADA (titer)	IDUA	Chim (% donor)	Doses missed
1	No	F	8	URD	13	0	WNL	79	0
2	Yes	M	33	UCB	5	0	WNL	99	1
3	Yes	F	35	UCB	5	400	50% of NL	77	0
4	Yes	M	16	UCB	8	100	WNL	81	11
5^a^	No	F	2	UCB	10	0	WNL	72	6
6^a^	No	M	32	RD	13	0	60% of NL	90	6
7	No	M	9	UCB	11	0	WNL	100	1
8	Yes	F	24	RD	10	0	50% of NL	100	1
9	No	M	31	RD	8	100	WNL	100	0
10^a^	Yes	M	7	RD	9	0	WNL	100	0

*Pt* participant, *ERT* enzyme replacement therapy, *HCT* hematopoietic cell transplantation, *m* months, *y* years, *ADA* antidrug antibodies at time of enrollment, *IDUA* iduronidase levels, *Chim* Chimerism, *URD* unrelated donor, *UCB* unrelated cord blood

^a^Developed high and persistent antidrug antibodies and neutralizing antibodies during the study

There were 23 historical controls who were 48% male (*n* = 11) with mean age of 9.3 ± 3.5 years (mean age in treated group was 9.2 ± 2.8 years). At the time of enrollment, the historic control group was on average 8.0 ± 3.6 years from HCT (7.6 ± 3.5 years from HCT for treated group). The average age at HCT was 15.4 ± 8.2 months in controls and 19.7 ± 12.7 months in the treated group. 39% vs. 50% received ERT peri-HCT in controls versus treated group, respectively. None were treated with ERT at the time of enrollment in either study.

### Efficacy outcomes

Due to missing data, outcome comparisons were preferentially made from baseline to 24 months, but if baseline and/or 24-month data were not available, the longest interval between measurements was reported, with a minimum requirement of 12 months. Missing data are indicated in each table.

#### Growth

**Fig. 1:**
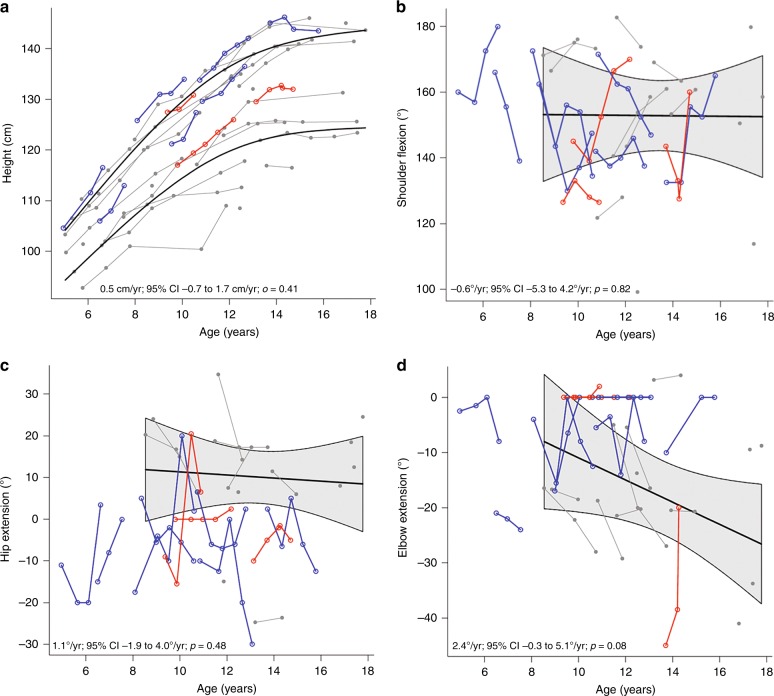
Comparison of change in **a** height and **b**−**d** range of motion in treated participants compared to historic MPS IH control data. Blue connected circles indicate participants who *did not* develop high antidrug antibodies during the study. Red connected squares indicate participants who *did* develop high antidrug antibodies with neutralizing antibodies during the study. Historic male and female control data are shown in gray and includes linear mean trajectory with 95%CI shown by shaded area

Change in growth velocity was determined by calculating the difference between baseline and month 24 growth velocities. Baseline growth velocity was calculated using the most recent measure done in clinical practice prior to study enrollment (median 322 days prior [range 188−489 days]), which was available in six of ten participants. One female participant was excluded from the within-group growth analyses due to a bone age of 14.5 years (i.e. closed growth plates). Change in growth velocity was −1.1 ± 3.8 cm/yr (range −7.0 cm/yr to +4.0 cm/yr). There was no statistically significant difference in comparison of the entire cohort in annualized growth velocity in treated patients versus historic controls (Fig. 1). However, the two participants under age 8 years old had a significant improvement in annualized growth velocity compared to 12 controls with the same age restriction (2.4 cm/yr; 95% CI 0.8−3.9 cm/yr; *p* = 0.002). This is supported by the fact that a younger age at study entry was associated with a better change in height (adjusted for age and sex) over the study (−0.17 SDS/year; 95% CI −0.34 to −0.01 SDS/year; *p* = 0.04]).

#### Fitness and 6MWT

For peak HR on the treadmill testing, the average change over 12–24 months was +23 ± 18 bpm (range −7 to +44 bpm). The average change in time to fatigue was 6.2 ± 5.0 min (range −0.8 to 17.2 min), and the average improvement in 6MWT over 18–24 months was 50 ± 92 m (range −104 to +264 m). Within the entire group, there was no significant association of change in 6MWT or time on treadmill with age at study entry.

#### Range of motion

Four of ten participants had improvements (defined for all joints as >5°) in left or right shoulder ROM, 3/10 had improvements in left or right elbow ROM, and 5/10 had improvements in left or right hip ROM (Table 2). Change in ROM was not different from historic controls (Fig. 1) and was not associated with age at study entry.

**Table 2:** Change in passive joint range-of-motion (ROM) over 12−24 months

Outcomes	Change over 12–24 months Mean ± SD (range)	>5° improvement in ROM *N*/Total (%)	>5° worsening in ROM *N*/Total (%)
Shoulder flexion—left, °	8 ± 33 (−44 to 72)^2^	4/8 (40)	3/8 (30)
Shoulder flexion—right, °	5 ± 24 (−32 to 36)^2^	4/8 (40)	3/8 (30)
Elbow extension—left, °	3 ± 9 (−12 to 16)^5^	3/5 (30)	1/5 (10)
Elbow extension—right, °	−1 ± 5 (−9 to 10)^5^	1/5 (10)	1/5 (10)
Hip extension—left, °	1 ± 14 (−15 to 22)^2^	4/8 (40)	3/8 (30)
Hip extension—right, °	0 ± 14 (−25 to 17)^2^	5/8 (50)	3/8 (30)

Superscript numbers indicated # missing data for either baseline or month 24 visit. Percent with improvement or worsening in ROM is calculated based on total number with available data

#### Muscle strength

Change in handgrip strength was +1.1 ± 1.9 kg (range −0.7 to 4.0 kg). There was no change in dominant leg muscle strength measured as peak torque per 100 lbs at 90 and 120 s. Neither change in handgrip strength nor in dominant leg muscle strength was associated with age at study entry.

#### Cardiac

Eight of ten subjects had both baseline and month 24 echo data. All had left ventricular end-diastolic diameters, posterior wall and septal thicknesses within 2 SD of normal and normal systolic cardiac function as evidenced by shortening fraction ([SF] mean 38%, range 34–52%). Measures of chamber dimensions, posterior and septal wall thicknesses, remained within 2 SD of normal for the duration of the study except for one subject whose left ventricular end-diastolic dimension increased from +1.9 to+3.2 SD and septal wall thickness from 0.4 to 2.6 SD; cardiac function remained normal at month 24 in all eight subjects (SF baseline = 38 ± 6.2%; SF month 24 = 37 ± 4%; *p* = 0.57). Of note, the subject who had an increase in left ventricular end-diastolic dimension and septal wall thickness also had the highest degree of mitral dysfunction within the series at baseline and the shortening fraction was hyper-dynamic (52%) at baseline, but normalized (39%) at month 24.

At baseline, mitral regurgitation was absent or trace/trivial in six subjects, mild in one and moderate in the eighth subject. Aortic regurgitation was absent or trace/trivial in five subjects and mild in three subjects. Aortic regurgitation tended to increase with increasing age. Neither mitral nor aortic stenosis was identified. For the eight subjects with both baseline and month 24 echo data, mitral regurgitation was unchanged in seven of eight subjects and increased from none to trivial in one subject over the course of the study. Aortic regurgitation was unchanged in five subjects but increased by one or more levels in three subjects (none to mild (1); none to trace/trivial (1); mild to moderate (1)).

#### Impact of anti-laronidase antibody status on outcomes

**Fig. 2:**
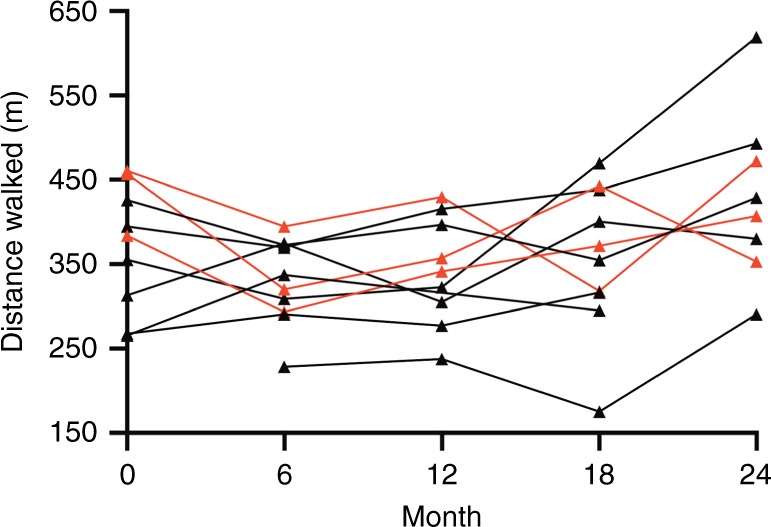
Comparison of change in 6MWT in ADA+ (red) versus ADA− (black)

Three participants (ID5, ID6, and ID10) developed persistently high antidrug antibody titers with neutralizing antibodies during the study (ADA+), with 40–98% uptake inhibition, that persisted for the duration of the study. Recognizing that antibody production may affect functional outcomes, we analyzed outcomes related to the presence (ADA+) or absence of persistent high neutralizing antibody titers (ADA−). Rate of change in all outcomes adjusted for age at enrollment and antibody status are shown in Table 3. The only clinically significant effect of ADA+status identified was a decrease in the 6MWT in comparison to the ADA− patients (−14 m; 95% CI: −28 to −1 m; *p* = 0.038 [Fig. 2]). And, when ADA+ patients were removed from the analysis, the ADA− group had a significant improvement in 6MWT over the study (82 m; 95% CI: 2−161 m; *p* = 0.045).

**Table 3:** Comparison of rates of change after adjusting for age at enrollment for outcomes in participants who developed antibodies compared to those who did not

Outcome	*N* obs, *N*	ADA+ minus ADA− (95% CI)	*p* value
6MWT, m	46, 10	**−14.35 (−27.91, −0.79)**	**0.038**
Left shoulder flexion, °	45, 10	**1.11 (0.14, 2.07)**	**0.024**
Right shoulder flexion, °	45, 10	1.06 (−0.08, 2.19)	0.068
Left elbow extension, °	41, 10	0.07 (−0.43, 0.57)	0.779
Right elbow extension, °	41, 10	0.01 (−0.48, 0.51)	0.959
Left hip extension, °	46, 10	0.28 (−0.39, 0.96)	0.407
Right hip extension, °	46, 10	0.56 (−0.19, 1.31)	0.144
Max HR treadmill, bpm	43, 10	0.16 (−0.79, 1.11)	0.744
Time on treadmill, min	38, 10	−0.11 (−0.43, 0.22)	0.517
Handgrip, kg	45, 10	0.01 (−0.18, 0.19)	0.944
Peak torque at 90°/s, Ft-lbs/100 lbs	38, 10	−0.27 (−0.82, 0.27)	0.322
Peak torque at 120°/s, Ft-lbs/100 lbs	38, 10	−0.16 (−0.66, 0.33)	0.524
Sitting height, cm	37, 10	−0.07 (−0.18, 0.04)	0.195
Arm span, cm	38, 10	−0.06 (−0.35, 0.23)	0.680
Standing height, cm	40, 10	0.01 (−0.18, 0.21)	0.884
Height SDS	40, 10	0.00 (−0.02, 0.03)	0.904

A negative coefficient means antibody positive participants had a more negative change in the outcome after adjustment for age at enrollment compared to antibody negative participants

Statistically significant p-values are bold <0.05

#### Global outcome assessment

**Fig. 3:**
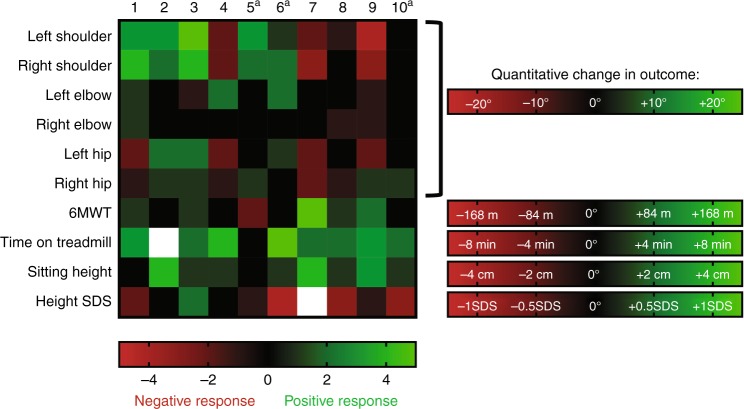
Heatmap of outcomes for each individual participant. Green indicates more positive response to treatment, red a more negative response to treatment, and white with X indicates missing data. A “^+^” indicates that the participant developed high antidrug antibodies with neutralizing antibodies during the study. 6MWT 6-min walk test, TM treadmill, SDS standard deviation score. 6MWT grading based on previously reported change in 6MWT with long-term laronidase treatment^33^

A heatmap indicating a positive response in green, no response in black, and a negative response in red is included to provide a global response to treatment (Fig. 3). Outcome-specific changes used to define each incremental change in the heatmap color are detailed in Figure 3 as well.

## Discussion

This study evaluated ERT augmentation in a post-HCT population with MPS IH. We found that ERT, 2 or more years after HCT in MPS IH, was safe and tolerable (rate of adherence to the schedule of weekly infusions of laronidase ranged from 79 to 100%). There was an improvement in growth velocity in the two youngest participants compared to historic controls, and an improvement in the 6MWT in participants who did not develop high, persistent, neutralizing antidrug antibodies. Cardiac echo parameters remained mostly unchanged throughout the study but mild progression of aortic valve regurgitation was seen in ~40% of subjects.

Severe limitations in joint ROM is present in most patients with MPS IH despite successful engraftment after HCT. In a prior study of such patients, an improvement from pre-HCT to post-HCT in knees, hips, and elbow ROM in 38–63% of patients followed over an average of 6.4 years after HCT was found.^37^ Our historic data on transplanted children with MPS IH who did not undergo long-term ERT augmentation indicate that shoulder flexion, elbow extension, and hip extension deteriorate slowly over time (Fig. 1). In the attenuated form of MPS I (MPS IA), joint stiffness improves with laronidase treatment but is not entirely alleviated.^31,38,39^ The longest study of joint outcomes in patients with MPS I has been in MPS IA where it is reported that after the same duration of therapy as our study (2 years) the average improvement in shoulder flexion on ERT was 29° (range 5°−50°).^39^ For MPS IH ROM after HCT, we know of only one publication on ROM response to a treatment besides HCT. In that publication, a patient with MPS IH was treated with adalimumab, an antiinflammatory medication used for juvenile idiopathic arthritis, for 16 weeks. There was a response in shoulder flexion of 20° and 49°, seen in the left and right shoulders respectively.^40^ We found much more variability than either of these studies. In our study the range of change in shoulder flexion was −44° to +72° over 2 years. This is likely related to the high degree of variability in the severity of joint disease in patients with MPS IH after HCT, and was similar in regards to our observances in elbow extension.

We obtained two measures of fitness during our study: the modified Balke treadmill test and the 6MWT. The measurement of peak oxygen uptake during treadmill testing is considered the best single physiologic indicator of an individual’s cardiopulmonary fitness and can also be used to monitor changes in cardiopulmonary fitness over time.^29,41^ Unfortunately, our participants were rarely able to tolerate the mask necessary for this measurement during the treadmill test, and so this outcome proved not feasible. They were, however, able to perform the 6MWT, on which MPS IA participants have previously shown improvements of 19.7 ± 68.6 m over 26 weeks^32^ and 31.7 ± 10.2 m over 2 years of laronidase ERT.^31^ We found a mean increase on the 6MWT that was 2.5 and 1.6 times these prior studies, but also had greater variability: 50 ± 92 m. The variability in response to therapy is likely due to a broad spectrum of physical impairment in MPS IH patients after HCT, other interventions such as surgical procedures, and variation in motivation.

Prior reports of ADA following IV laronidase found a high incidence of the development of ADA that appear to have a negative impact of biochemical measures of disease activity.^28,42,43^ For example, in one study of MPS IH patients treated before and during HCT, 50% of patients developed ADA with both catalytic enzyme inhibition and uptake inhibition of catalytically active enzyme, but became immune tolerant 26–137 days after HCT.^42^ However, there was a negative impact of ADA on the ratio of dermatan sulfate to chondroitin sulfate in the six patients studied.^42^ Others have found that laronidase ADA correlate with worse sleep disordered breathing.^28^ In our study, patients who did not develop high ADA had a significant improvement in their 6MWT over the course of the study; however, patients with ADA did not improve on their 6MWT. This clinical outcome data seems to support previously published data on the negative impact of ADA on biochemical outcomes. However, the question remains as to whether patients with MPS IH treated with ERT after HCT will become immune tolerant over time, as has been reported,^39,42^ or whether ADA persist and negate the positive impact of enzyme secreted by donor cells.

Progressively worsening short stature has been reported by us and others in patients with MPS IH after HCT.^3,5^ On average, after HCT patients with MPS IH lose approximately 0.5 height SDS every 2 years; however, this varies slightly with age and post-HCT leukocyte IDUA levels.^3,5^ Five of our participants had a change in height SDS of better than the expected loss of 0.5 SDS every 2 years, and three of our study patients had a positive increase in height SDS of 0.1, 0.4, and 0.7. The improvement in annualized growth velocity in our treated group versus historic controls was 0.9 cm/yr (95% CI: −0.4 to 2.2 cm/yr; *p* = 0.16), with the greatest increase in comparative growth velocity in the youngest two participants (Fig. 1). The clinical significance of these improvements is unclear based on the limited sample size, but suggests that additional study would be useful to determine if this finding is observed in a larger group of younger patients.

Cardiac echo structural and functional parameters were within normal limits in all subjects and cardiac valve regurgitation was absent or trivial in most subjects at the initiation of ERT. The structural and functional parameters remained normal after 24 months of ERT in all except one subject who began the study with moderate mitral regurgitation. Progression of aortic valve regurgitation has previously been reported as a long-term finding in MPS I patients after both HCT^44^ and enzyme replacement therapy.^45^

Although this study was primarily designed to understand safety and tolerability, the small sample size, which is an inherent limitation in rare disease research, restricts the number of factors that can be controlled when modeling outcomes, therefore requiring a conservative approach in drawing definitive conclusions. It is also possible that, given the small sample size, a longer study is needed to see statistically significant, and clinically meaningful, improvements in outcomes in this small group. Our study highlights the challenges in measuring the impact of disease burden in this pediatric population due to a variety of reasons, such as an inability to understand test directions, maintenance of motivation, and other factors such as disease and/or surgeries. For instance, we determined that assessment of cardiopulmonary fitness (VO_2_ peak from the Modified Balke Treadmill test) was not feasible in our cohort due to an inability to wear the required mask while walking on the treadmill. Despite these limitations, this study provides (1) valuable insight into how a future study should be designed, considering the physical and cognitive limitations of the population, and (2) pilot data to adequately power a future study in order to draw definitive conclusions about the effects of laronidase in patients with MPS IH after HCT.

In conclusion, enzyme augmentation with laronidase 0.58 mg i.v. weekly is well tolerated in posttransplantation MPS IH patients. However, we cannot make conclusions on the usefulness of ERT in patients with MPS IH after HCT at this time due to the limitations described above. Additional testing is needed. One concerning finding however, is that we observed the development of antidrug antibodies in several patients, which was not anticipated, as the hematopoietic cells of these patients are endogenously producing enzyme. In addition, there is a suggestion that antibody generation may be clinically important, as an improvement was noted in the 6MWT in the individuals who did not develop high ADA levels. An improvement in growth rates were also seen, but only for the younger participants. If enzyme is used after transplantation, monitoring patients for the development of antibody would be important, as at least in the case of the 6MWT, it may be clinically relevant.
